# A Concise and Practical Treatise on the Principal Diseases of the Air-Passages, Lungs, and Pleura

**Published:** 1842-01

**Authors:** 


Art. VII.—*A Concise and Practical Treatise on the principal Diseases of the Air-passages, Lungs, and Pleura.* By A. Catherwood, m.d. c.m.—*London*, 1841. 8vo, pp. 208.

We know not where Dr. Catherwood can have learned that "a concise treatise on the diseases of the lungs, &c. has long been a disideratum;" and we are most certain that the work he has produced could never "stop the gap in the medical literature of the country," if it really existed. He has, he says, written for "the public generally" as well as the profession, having "endeavoured, in every instance, to adapt the language to the comprehension of the non-medical reader." Now Dr. Catherwood must have formed an exalted estimate of the knowledge of his non-medical readers, since he quotes German by the page, and Italian and Latin occasionally, without remorse, and without translation. To be sure, the quotations are all about very simple matters, and are certainly not worth translating; but we may be allowed to wonder why they were disturbed in their native places. For instance, he tells us, *in German*, the remarkable novelty, that a certain Dr. C. A. W. Berends "has correctly observed" that the palms and soles particularly are hot in hectic fever! Sometimes, however, Dr. Catherwood condescends to translate—as where he tells us (p. 159) that in certain cases "*das geräusch des gesprungenen topfes* of the *German* writers is heard on percussion," which "*sound of the boiling pot* can be imitated," &c. Now, surely Dr. Catherwood cannot be ignorant that this particular sound does not belong to the *Germans* at all, but to the *French* (bruit de pot felé), and that both the original French expression and its German translation mean a *cracked* not a *boiling* pot. But we could easily pardon immature taste parading unnecessary quotations, and many other defects of style and manner wherewith the book is rife, if it contained anything that all the world did not know before; or even if it presented what was known, in a more useful or more attractive form, for the benefit of either the profession or the public. We are sorry to say, however, that this is not the case, and we end as we began by wondering what induced Dr. Catherwood to risk his professional credit in so rash a volume as the present.

